# High expression of the human equilibrative nucleoside transporter 1 gene predicts a good response to decitabine in patients with myelodysplastic syndrome

**DOI:** 10.1186/s12967-016-0817-9

**Published:** 2016-03-05

**Authors:** Lingyun Wu, Wenhui Shi, Xiao Li, Chunkang Chang, Feng Xu, Qi He, Dong Wu, Jiying Su, Liyu Zhou, Luxi Song, Chao Xiao, Zheng Zhang

**Affiliations:** Department of Hematology, Shanghai Jiao Tong University Affiliated Sixth People’s Hospital, Shanghai, 200233 China

**Keywords:** Myelodysplastic syndrome, Decitabine, Human equilibrative nucleoside transporter 1, Long interspersed nuclear element

## Abstract

**Background:**

Despite the efficacy of decitabine treatment in myelodysplastic syndrome (MDS), no definite predictor of response is known. In this study, we investigated whether the expression levels of human equilibrative nucleoside transporter 1 (*hENT1*), *hENT2*, deoxycytidine kinase (*DCK*) and cytidine deaminase (*CDA*) genes could predict response to decitabine in MDS.

**Methods:**

We performed quantitative real-time PCR in marrow mononuclear cells to examine the expression of *hENT1*, *hENT2*, *DCK*, and *CDA* prior to therapy in 98 MDS patients initially treated with decitabine. Response and overall survival of patients treated with decitabine were analyzed according to gene expression levels. *HENT1* knockdown was performed by shRNA in the SKM-1 cell line, and the effect of this on the demethylation ability of decitabine on long interspersed nucleotide element 1 (LINE1) was investigated.

**Results:**

Patients responding to decitabine presented with significantly higher *hENT1* expression levels than non-responders (*p* = 0.004). Overall response, complete response, and cytogenetic complete response rate in patients with high *hENT1* expression (79.4, 41.3, and 43.8 %) were significantly higher than those in patients with low *hENT1* expression (48.6, 20.0, and 5.9 %, respectively) (*p* = 0.004, 0.033, and 0.006, respectively). In higher-risk MDS, patients with high *hENT1* expression showed prolonged survival compared with those with low *hENT1* expression (22.0 vs 14.0 months; *p* = 0.027). However, the expression levels of *hENT2*, *DCK*, and *CDA* did not affect response rate. Knockdown of *hENT1* in SKM-1 cells weakened the demethylation effect on LINE1 induced by decitabine.

**Conclusions:**

High expression of *hENT1* appears to predict a good response to decitabine and a prolonged survival in higher-risk MDS patients treated with decitabine. *HENT1* expression knockdown weakens the demethylation effect of decitabine.

**Electronic supplementary material:**

The online version of this article (doi:10.1186/s12967-016-0817-9) contains supplementary material, which is available to authorized users.

## Background

Myelodysplastic syndromes (MDS) are recognized as among the most common hematological neoplasms [1], comprising a heterogeneous group of clonal hematopoietic stem/progenitor cell disorders characterized by ineffective hematopoiesis, dysplastic cell morphology, and the potential for clonal evolution [2]. Approximately 50–60 % MDS patients have abnormal karyotypes, frequently with 5q-, +8, −7(q) or 20q- [1]. Decitabine is one of the drugs approved for treatment of MDS, and low-dose decitabine has been proven to be effective for some MDS patients [3, 4]. However, a cohort of patients do not respond to this treatment initially [4, 5], and there are almost no effective factors predicting which patients will respond to decitabine before starting the therapy.

It is likely that genetic variability of key enzymes in decitabine transport and metabolism may have an impact on the treatment response of decitabine. Thus, the expression levels of genes related to decitabine transport [including human equilibrative nucleoside transporter 1 (*hENT1*) and *hENT2*], and metabolism [including deoxycytidine kinase (*DCK*) and cytidine deaminase (*CDA*)] may influence the therapeutic effect of the drug.

Because the biochemical targets of decitabine are intracellular, the mandatory first step in the production of cytotoxicity is permeation across the cell membrane. Decitabine and physiologic nucleosides are hydrophilic, and diffusion through the cell membrane lipid layer is slow. Therefore, efficient cellular uptake requires the presence of specialized integral membrane nucleoside transporter proteins [6]. Two general processes of nucleoside transport have been identified: the equilibrative bi-directional facilitators (hENT1 and 2) and the concentrative sodium/nucleoside symporters hCNT1 and 3. The major routes for transporting decitabine are hENT1 and hENT2, and, to a lesser extent, hCNT1 and hCNT3 [6]. When transporting into the cell, decitabine is phosphorylated into monophosphorylated derivative 5-azadCMPby deoxycytidine (DCK). Then, 5-aza-dCMP is phosphorylated to 5-aza-dCTP, which is the active form of decitabine and incorporated into DNA, where it acts by demethylation. Catabolizing enzymes such as cytidine deaminase (CDA) can catalyze decitabine metabolites to uridine and deoxyuridine, therefore decreasing the amount of 5-aza-dCTP that can be formed.

In solid tumors such as pancreatic ductal adenocarcinoma, hENT1 expression was used to identify subgroups of patients with different disease progression risk, while DCK expression was shown to be most relevant to mortality risk [7]. In cancer cell lines, those most resistant to decitabine had a combination of low expression of DCK, hENT1 and high CDA expression [8]. Furthermore, a high CDA/DCK ratio has been reported to predict response to decitabine [9]. A recent study by Wu et al. [10] showed that high hENT1 expression is a predictor for response to decitabine in MDS. However, the samples involved in their study were insufficiently large, and the effect of hENT1 expression on patient survival was not addressed. Furthermore, to our knowledge, no in vitro studies have shown the direct effect of hENT1 expression on the demethylation of decitabine in MDS cells.

Here, we enrolled a total of 98 patients treated with decitabine to further determine whether hENT1, hENT2, DCK, and CDA can affect the response and prognosis in MDS patients treated with decitabine. We also investigated the alteration of hypomethylation and the subsequent biological actions induced by decitabine in the SKM-1 cell line following knockdown of *hENT1* expression.

## Methods

### Patients

All patients enrolled in this study had been diagnosed with MDS at our department according to French–American–British (FAB)/World Health Organization (WHO) classification criteria [11, 12]. Bone marrow (BM) samples were obtained at diagnosis of MDS before the initiation of any treatment between September 2009 and December 2013 following approval by the local ethics committee and according to institutional guidelines. Each of the 98 MDS patients treated with decitabine provided written informed consent. The characteristics of the MDS patients are listed in Table 1. As of December 2014, the median follow-up period was 24 months (range 2–62 months). This study was approved by the Ethics Committee of Shanghai Jiao Tong University Affiliated Sixth People’s Hospital.

**Table 1:** Characteristics of the 98 MDS patients treated with decitabine

Characteristics	Category
Median age (range)	62 (20–82)
Sex, n (%)	
Male	69 (70)
Female	29 (30)
WHO/FAB classification, n (%)	
RCMD	19 (20)
RAS	2 (3)
RAEB-1	25 (22)
RAEB-2	29 (30)
RAEB-t	14 (17)
CMML	9 (8)
Karyotypes, n(%)	
Normal	49 (50)
+8	7 (7)
−7/7q-	9 (9)
−5/5q-	2 (2)
20q-	5 (5)
Complex	13 (13)
Others	13 (13)
International prognostic scoring system, n (%)	
Low	4 (4)
Intermediate-1	33 (34)
Intermediate-2	34 (35)
High	27 (27)

*MDS* myelodysplastic syndrome, *FAB* French-American-British, *WHO* world health organization, *RCMD* refractory cytopenia with multilineage dysplasia, *RAS* refractory anemia with ringed sideroblasts, *RAEB* refractory anemia with excess of blasts, *RAEB-t* refractory anemia with excess of blasts in transformation, *CMML* chronic myelomonocytic leukemia

### Real-time quantitative polymerase chain reaction

Total RNA was extracted from 2 ml of BM mononuclear cells (BMNCs) using the RNeasy Mini Kit (QIAGEN, Hilden, Germany) according to the manufacturer’s instructions. cDNA was synthesized by random priming from 10 µL of total RNA using the RevertAid™ First Strand cDNA Synthesis Kit (Fermentas, Burlington, Canada) according to the manufacturer’s instructions. PCR was performed in a fluorescent quantitation PCR cycler (LightCycler, Roche, Switzerland) in a final volume of 10 μL, including 1 µL of cDNA, 0.5 μM of each primer, 4 mM MgCl_2_, and 2 μL of the supplied enzyme mix containing the reaction buffer, FastStart Taq DNA polymerase, and DNA double-strand-specific SYBR Green I dye for PCR product detection. Primer sequences were as follows: hENT1 (NM_001078177.1): 5′-TCTCCAACTCTCAGCCCACCAA-3′ (sense) and 5′-CCTGCGATGCTGGACTTGACCT-3′ (antisense); hENT2 (NM_001532.2): 5′-ACCATGCCCTCCACCTACAG-3′ (sense), 5′-GGGCCTGGGATGATTTATTG-3′ (antisense); DCK (NM_000788.2): 5′-GGCCGCCACAAGACTAAGGA-3′ (sense) and 5′-CACCATCTGGCAACAGGTTCA-3′ (antisense); and CDA (NM_001785.2): 5′-CCTGCAGGCAAGTCATGAGAG-3′ (sense); 5′-ACCATCCGGCTTGGTCATGTA-3′ (antisense). The primer sequences for *GAPDH* were as follows: 5′-GCACCGTCAAGGCTGAGAAC-3′ (sense) and 5′-GTGGTGAAGACGCCAGTGGA-3′ (antisense). PCR conditions were as follows: preincubation at 95 °C for 30 s, then 40 cycles of 95 °C for 15 s, 62 °C for 30 s, and 72 °C for 30 s. The threshold cycle (Ct) value was subsequently determined, and the relative quantification of mRNA expression was calculated using the comparative Ct method.

The relative quantification value of the target, which was normalized to that of an endogenous control (*GAPDH*) and relative to that of a calibrator (the mean expression level of normal controls), was expressed as 2^−ΔΔCt^ (fold difference) where ΔCt = Ct of the target gene-Ct of the endogenous control gene (GAPDH) and ΔΔCt = ΔCt of the samples for the target gene −ΔCt of the calibrator for the target gene [13]. To analyze the characteristics of target gene expression levels in MDS treated with decitabine, the mean expression level of target genes in BM from 20 normal donors (median age, 60 years; male: female 2:1) was used as a control. Based on control mean expression levels of each gene, we divided the enrolled patients into two groups: a high gene expression group with 2^−ΔΔCt^ values above mean levels, and a low gene expression group with 2^−ΔΔCt^ values below mean levels.

### Decitabine treatment

All patients were treated with decitabine (Johnson and Johnson Inc., New Brunswick, NJ), which was administered at a dose of 20 mg/m^2^ by continuous intravenous infusion for 1 h, and repeated daily for 5 days. The cycle was repeated every 4 weeks, depending on each patient’s recovery from myelosuppression. BM examinations were performed 4 weeks after decitabine treatment was completed to evaluate the response. The final treatment response was assessed after at least four cycles of decitabine therapy, except for patients who discontinued therapy because of disease progression when receiving fewer than four cycles of therapy.

### Responses to decitabine therapy and overall survival

The primary observation parameter was the overall response rate (OR), which included the rates of complete remission (CR), marrow CR (mCR), hematologic improvements (HI), and cytogenetic CR (CCR) (only in patients with abnormal karyotypes before therapy). The treatment response was assessed using modified International Working Group (IWG 2006) response criteria [14]. Overall survival (OS) was calculated from the initial date of decitabine treatment to the date of death from any cause.

### Cell line and culture

Decitabine was purchased from Sigma-Aldrich (St Louis, MO), dissolved in dimethyl sulfoxide (DMSO; Sigma-Aldrich) at 10 mM, and stored at −20 °C. SKM-1 cells (Health Science Research Resources Bank, Osaka, Japan) were grown in RPMI-1640 medium (Gibco, Grand Island, NY) containing 10 % heat-inactivated fetal bovine serum, 100 IU/ml penicillin, and 100 µg/ml streptomycin in a humidified atmosphere of 5 % CO_2_ at 37 °C. Cells were seeded in 12-well plates at a density of 5 × 10^5^ cells in 2 ml culture medium and treated with DMSO or decitabine every 24 h. After 24, 48, and 72 h, cells were harvested and DNA was isolated using DNA reagent (QIAGEN, Hilden, Germany) according to the manufacturer’s instructions. As a solvent control, DMSO was added to a final concentration of 0.01 %.

### Lentivirus-delivered small interfering (si)RNA gene knockdown

The siRNA sequence for hENT1 knockdown was 5′- ACCAATGAAAGCCACTCTA-3′, and the scrambled control siRNA sequence was 5′-TTCTCCGAACGTGTCACGT-3′. The siRNA sequences were cloned into the pLSLG lentiviral vector. This and the help vector (pLV-HELP) were transfected into 293FT cells for viral packaging. Virus was then collected for the infection of SKM-1 cells in the presence of 5 μg/ml polybrene (Genechem, Shanghai, China). After 4 days of culture, cells were used for further experiments.

### Pyrosequencing to measure LINE-1 methylation

Genomic DNA was extracted using DNeasy Blood & Tissue Kit (QIAGEN, Hilden, Germany) according to the manufacturer’s instructions and then modified by treatment with sodium bisulfite using the Zymo EZ DNA Methylation kit (Zymo Research, Irvine, CA) following the manufacturer’s protocol. A modified method of PCR-based pyrosequencing was performed to quantify methylation of LINE-1 repetitive elements, as previously described [15]. The primers used in PCR were: forward primer (5′-TTTTTTGAGTTAGGTGTGGGATA-3′) and biotinylated reverse primer (Biotin-AAAAATCAAAAAATTCCCTTTCC-3′). PCR cycling conditions were: 45 cycles of 95 °C for 15 s, 50 °C for 20 s, and 72 °C for 30 s. The PCR product was bound to streptavidin Sepharose beads (Amersham Biosciences, Uppsala, Sweden) and then purified. The LINE-1-S3 pyrosequencing primer (5′-GGGTGGGAGTGAT-3′) was annealed to the purified single-stranded PCR product. Pyrosequencing was performed using the PSQ HS 96 Pyrosequencing System. The average relative amount of cytosine in the three CpG sites was used as the overall LINE-1 methylation level.

### Statistical methods

Statistical analyses were performed using the Statistical Package for Social Sciences (SPSS 16.0; SPSS Inc., Chicago, IL). Two independent sample populations were compared using the Student’s *t* test. The Pearson χ^2^ or Fisher’s exact test was applied to compare the enumeration data between groups. The Kaplan–Meier method was used to estimate median OS. The log-rank test was used to compare Kaplan–Meier survival estimates between groups. Multivariate Cox proportional hazards models were used to calculate the hazard ratios and 95 % confidence intervals of the associations between risk factors and survival. *p* values <0.05 were considered statistically significant.

## Results

### Patient characteristics

A total of 98 patients were enrolled in this study. The median patient age was 62 years (range 20–82 years), and the male to female ratio was 2.38:1 (69:29). All patients in this study were diagnosed with de novo MDS. The FAB/WHO disease sub-types at study entry were refractory cytopenia with multilineage dysplasia (RCMD; n = 19), refractory anemia with ringed sideroblasts (RAS; n = 2), refractory anemia with excess blasts (RAEB-1; n = 25 and RAEB-2; n = 29), refractory anemia with excess blasts in transformation (RAEB-t; n = 14), and chronic myelomonocytic leukemia (CMML) (n = 9). The International Prognostic Scoring System (IPSS) [16] risk category was low in four, intermediate (INT)-1 in 33, INT-2 in 34, and high risk in 27 patients. A total of 49 patients (50.0 %) with abnormal karyotypes were determined by G-binding analysis [17]. Patient characteristics are listed in Table 1. The median number of decitabine therapy cycles was four (range 2–10). Eight patients discontinued treatment after two cycles of decitabine therapy because of disease progression. All patients were evaluable.

### *HENT1* expression levels were significantly elevated in responders to decitabine therapy

**Fig. 1:**
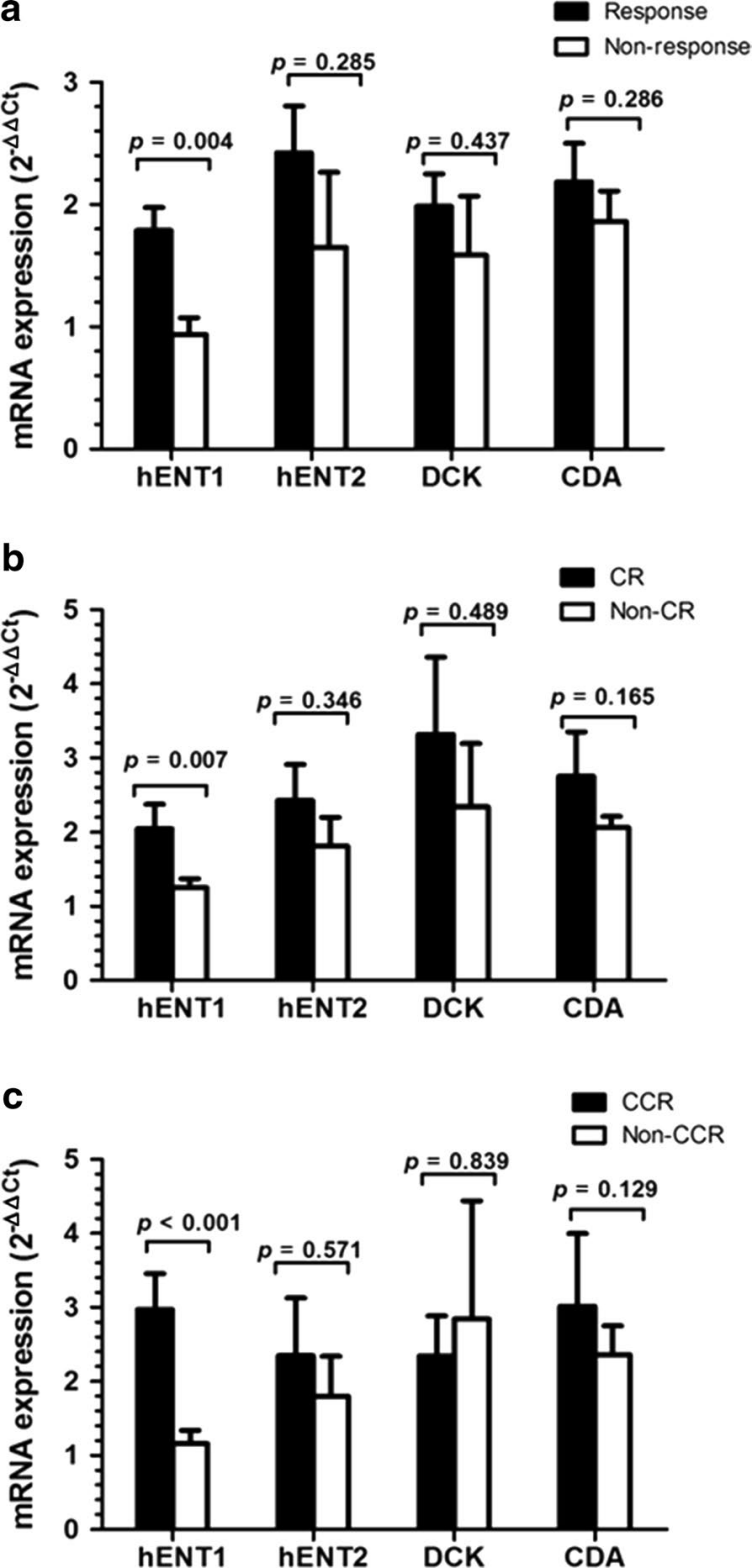
Comparison of *hENT1*, *hENT2*, *DCK*, and *CDA* expression levels between responders and non-responders; CR and non-CR; CCR and non-CCR. **a** Increased *hENT1* in responders compared to non-responders. *hENT2*, *DCK*, and *CDA* mRNA expression levels did not differ between responders and non-responders. **b** Elevated *hENT1* level in CR patients compared to non-CR patients. No significant difference of *hENT2*, *DCK*, and *CDA* mRNA level between CR and non-CR patients. **c** Increased *hENT1* rather than *hENT2*, *DCK*, or *CDA* level in CCR patients compared to non-CCR patients

Among the 98 MDS patients, 67 (68.4 %) achieved a response, including 33 (33.7 %) with CR, 11 (11.2 %) with mCR only, 15 (15.3 %) with HI only, and eight (8.2 %) with mCR with HI. Thirty-one (31.6 %) patients showed no response to decitabine therapy. Among the 49 patients with abnormal karyotypes, 15 (30.6 %) reached CCR. Patients responding to decitabine showed significantly increased mean *hENT1* expression levels compared with non-responders (1.79 ± 1.53-fold vs 0.94 ± 0.77-fold, *p* = 0.004) (Fig. 1a). Patients who achieved CR with decitabine also showed increased *hENT1* expression levels compared with those who did not achieve CR (2.05 ± 1.86-fold vs 1.25 ± 1.00-fold, *p* = 0.007) (Fig. 1b). Increased *hENT1* expression was also detected in patients who achieved CCR when compared with those who did not (2.97 ± 1.87-fold vs 1.16 ± 1.05-fold, *p* < 0.001) (Fig. 1c). *HENT1* expression level also increased in patients achieved mCR or HI when compared to that in NR patients (Additional file 1). Expression levels of *hENT2*, *DCK*, and *CDA* did not affect response including (OR, CR, CCR, mCR or HI) rate in MDS patients treated with decitabine (Fig. 1a–c; Additional file 1a, b).

### High expression of *hENT1* confers response to decitabine therapy

The mean expression level of *hENT1*, *hENT2*, *DCK*, and *CDA* of 20 normal donors was used as a control. Compared with these, a total of 63 patients (64.3 %) showed high *hENT1* expression and 35 (35.7 %) showed low *hENT1* expression. Among the 63 high *hENT1* expression patients, 50 (79.4 %) achieved a response, which was a significantly higher proportion than the 17/35 (48.6 %) low *hENT1* expression patients (*p* = 0.004). Twenty-six of 63 (41.3 %) *hENT1* high expression patients achieved CR, which was significantly higher than that of low *hENT1* expression patients (7/35, 20.0 %) (*p* = 0.033). There was no difference in mCR or HI rate between the high and low *hENT1* expression groups.

**Fig. 2:**
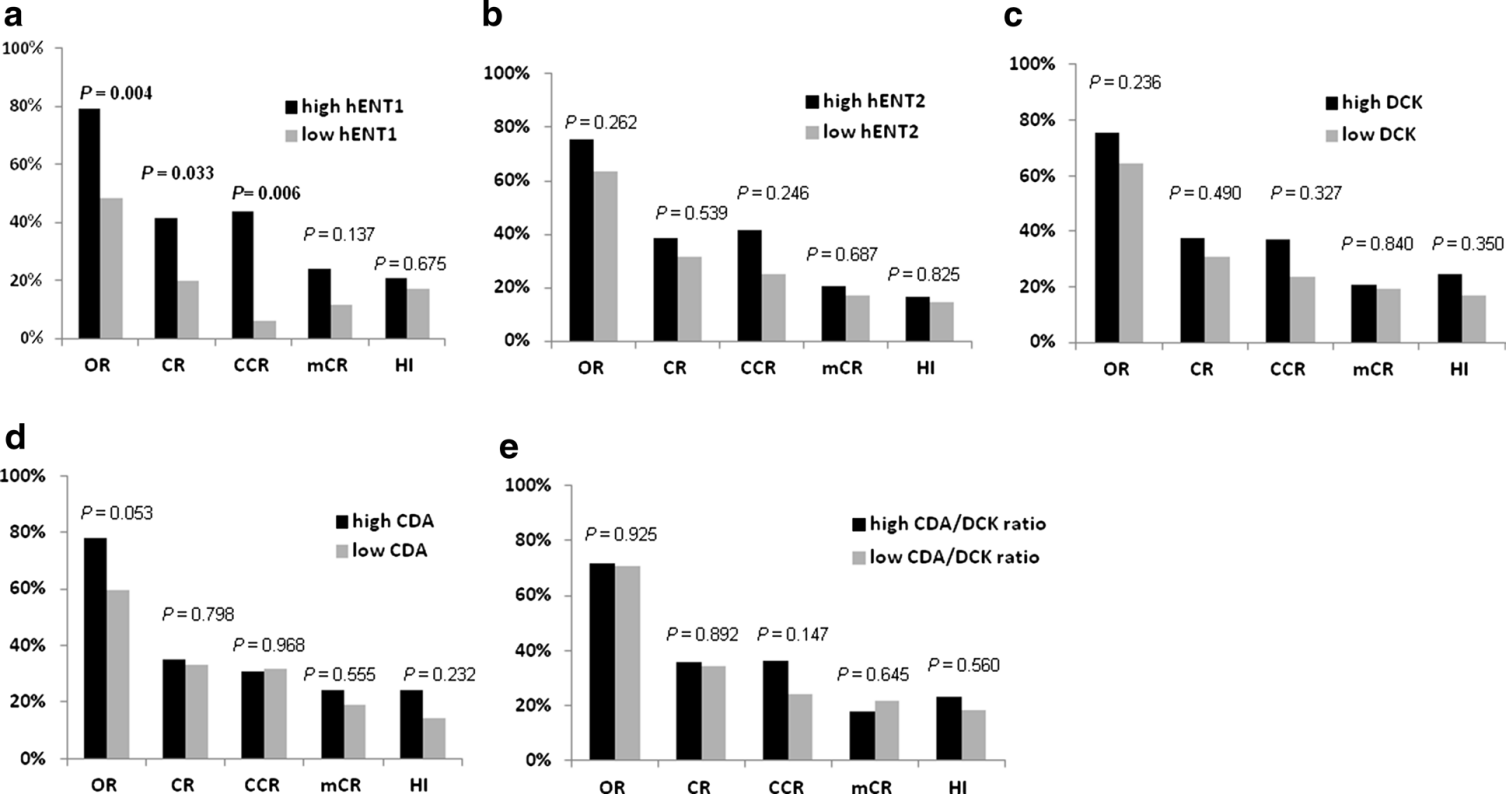
Comparison of response rate in patients treated with decitabine between those with **a** high and low *hENT1* expression, **b** high and low *hENT2* expression, **c** high and low *DCK* expression, **d** high and low *CDA* expression, and **e** high and low *CDA*/*DCK* ratio

Among the 49 patients with abnormal karyotype, the CCR rate was significantly higher in the *hENT1* high expression group (14/32, 43.8 %) than the low *hENT1* expression group (1/17, 5.9 %) (*p* = 0.006) (Fig. 2a). There was no significant difference in OR, CR, CCR, mCR, or HI between patients with high and low *hENT2* expression (Fig. 2b), high and low *DCK* (Fig. 2c), high and low *CDA* (Fig. 2d), or between the high and low *CDA*/*DCK* ratio (Fig. 2e).

### Fewer treatment cycles were needed for patients with high *hENT1* expression to achieve CR

We recorded the number of cycles needed for patients treated with decitabine to achieve CR. The median number of administered cycles was three (range 1–4) for all patients who achieved CR. For patients with high *hENT1* expression levels, significantly fewer cycles (median, two (range 1–4) cycles) were needed to achieve CR compared with low *hENT1* expression patients (median, three (range 2–4) cycles) (*p* = 0.035).

### High *hENT1* expression predicted prolonged survival in higher-risk MDS patients receiving decitabine treatment

**Fig. 3:**
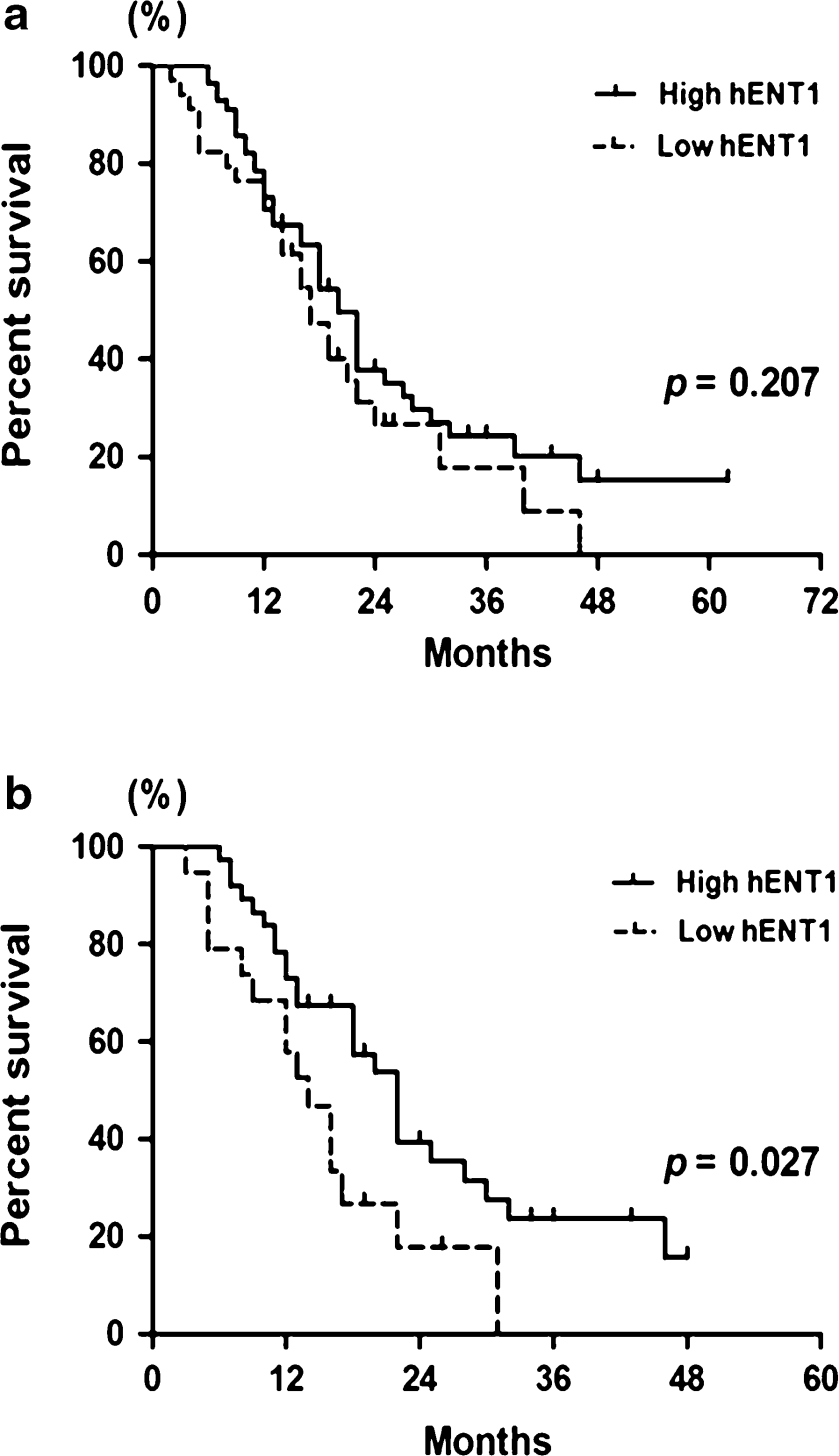
Comparison of overall survival of patients with high and low *hENT1* expression treated with decitabine. **a** In all MDS patients, the median overall survival time was not significantly different between high *hENT1* patients and low *hENT1* patients. **b** In the higher-risk patients, a prolonged overall survival time was observed in patients with high *hENT1* compared to those with low *hENT1*

During a median follow-up period of 24.0 months (range 2–62 months), the median OS of 98 patients was 18.0 months [95 % confidence interval (CI): 14.8–21.1 months]. The median OS between all MDS patients with high and low *hENT1* expression was not significantly different (23.0 vs 17.0 months; *p* = 0.207) (Fig. 3a). However, among the 61 higher-risk (IPSS int-2 or high-risk) patients, the median OS of patients with high *hENT1* expression was significantly longer than that of low *hENT1* expression patients (22.0 vs 14.0 months; *p* = 0.027) (Fig. 3b). Univariate analysis showed *hENT1* was a prognosis predictor in higher risk MDS patients treated with decitabine (*p* = 0.027). Furthermore, multivariate Cox analyses were conducted by integrating several risk factors including hemoglobin, platelet, BM blast, *hENT1 expression level.* In the multivariate analyses, low *hENT1* [hazard ration (HR) = 2.363; 95 % confidence interval (CI) = 1.174–4.757; Likelihood ratio test, *p* = 0.031] appeared to be independent prognostic markers of adverse events in higher risk MDS treated with decitabine (Table 2).

**Table 2:** Univariate and Multivariate analysis for survival in higher risk MDS patients treated with decitabine

Criterion	Univariate P value	Multivariate P value	HR	95.0 % CI for HR
Hemoglobin (>80 g/L vs ≤80 g/L)	0.010	0.026	2.023	1.177–4.112
Platelet (>50 × 10^9^/L vs ≤50 × 10^9^/L)	0.008	0.022	2.134	1.113–4.091
Bone marrow blasts percentage (5–10/10–20/>20 %)	0.010	0.041	1.522	1.017–2.277
*hENT1* expression (high vs low)	0.027	0.031	2.363	1.174–4.757
Age (>60 years vs ≤60 years)	0.796	–	–	–
Gender (male vs female)	0.569	–	–	–
Neutrophils (>1.0 × 10^9^/L vs ≤1.0 × 10^9^/L)	0.212	–	–	–
Karyotype (low/intermediate/high)	0.318	–	–	–
*hENT2* expression (high vs low)	0.201	–	–	–
*DCK* expression (high vs low)	0.203	–	–	–
*CDA* expression (high vs low)	0.411	–	–	–

### Lower expression of *hENT1* in SKM-1 cells reduces the demethylation effect of decitabine

**Fig. 4:**
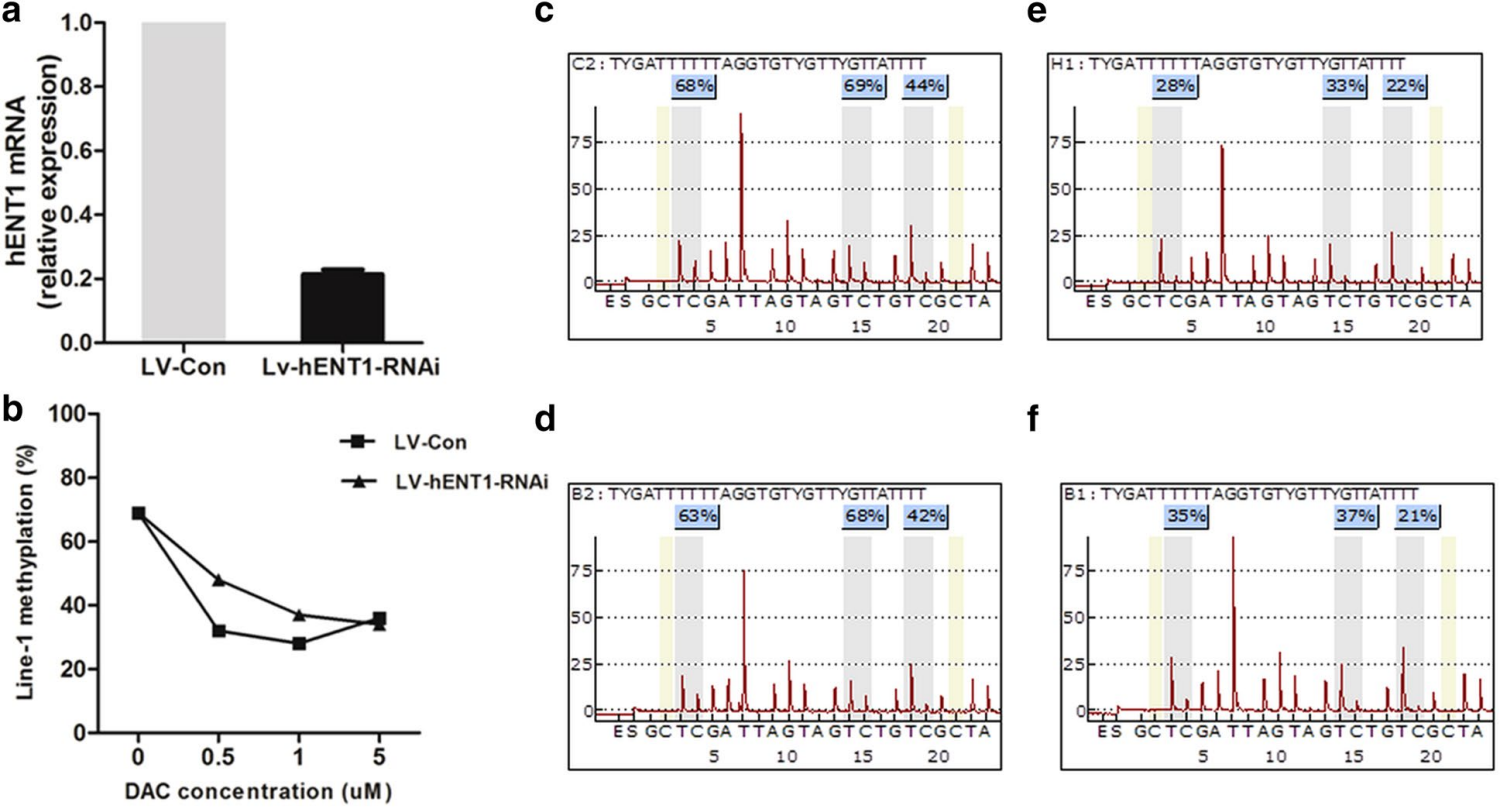
LINE-1 methylation level before and after decitabine treatment in hENT1-siRNA and control SKM-1 cells. **a** *hENT1* mRNA expression in SKM-1 cells transfected with hENT1-siRNA-lentivirus and control lentivirus. **b** LINE-1 methylation levels changed with different concentrations of decitabine. **c**–**f** A pyrosequencing assay was used to measure LINE-1 methylation before decitabine treatment in control lentivirus-transfected (**c**) and hENT1-siRNA cells (**d**). LINE-1 methylation was also measured after 1 µM decitabine treatment in control (**e**) and hENT1-siRNA cells (**f**)

HENT1 mRNA expression levels were significantly decreased in hENT1-siRNA-lentivirus-transfected SKM-1 cells compared with control lentivirus-transfected SKM-1 cells (Fig. 4a). Before decitabine treatment, the LINE-1 methylation status did not differ significantly between hENT1-knockdown and control cells. However, after decitabine treatment (at final concentrations of 0.5, 1.0, and 5.0 µmol/l), LINE-1 methylation levels were lowered in both hENT1-knockdown and control cells. The lowest methylation status was achieved following treatment with 1.0 µmol/l decitabine in both groups of cells. However, compared with the control group, the demethylation effect of decitabine was significantly weakened in hENT1-knockdown SKM-1 cells (*p* = 0.0026) (Fig. 4b–f). The biggest gap of demethylation effect between the two groups was observed at a decitabine treatment of 0.5 µmol/l.

## Discussion

Mechanisms of in vivo resistance to nucleoside analogs are complex and remain unresolved. One possibility may result from the presence of insufficient intracellular triphosphate, as seen for some nucleic acid analogs such as cytarabine and fludarabine [18].

Decitabine is administered to patients without knowledge of their genetic background, which may affect drug efficacy. The primary mechanism for insufficient intracellular concentrations of decitabine may result from a combination of multiple factors including inadequate uptake through membrane transporters, DCK deficiency, increased CDA, or high dNTP pools. HENT1 is the major membrane transporter for decitabine transporting into cell, which can affect the intracellular drug concentration and thus the efficacy of decitabine [19]. Studies both in cancer cell lines [8] and in MDS patients [9, 10] showed that resistant to decitabine had a low hENT1. In accordance with these findings, our results revealed significantly higher *hENT1* expression levels in decitabine responders compared with non-responders. Moreover, in CR patients, even higher *hENT1* levels were observed compared with non-CR patients. In patients with high *hENT1* expression, significantly higher response rate and CR rate was observed, compared with those of low *hENT1* expression patients. Therefore, high *hENT1* expression predicts response to decitabine in MDS patients and a higher possibility to achieve CR.

For MDS patients with abnormal karyotypes, CCR means the deeper remission and prolonged relapse-free survival [20]. To our best knowledge, there is no effect indictor for predicting CCR in MDS patients received decitabine therapy. Of note, in the current study, results showed that patients achieved CCR had a significantly higher *hENT1* expression, compared with non-CCR cases (2.97 ± 1.87-fold vs 1.16 ± 1.05-fold, *p* < 0.001). Among the 15 CCR patients, 14 presented with high *hENT1* expression, and only one showed low *hENT1* levels. Therefore, a high *hENT1* level is a critical factor to achieve CCR for MDS patients treated with decitabine.

A high *CDA* or *CDA*/*DCK* ratio has previously been reported to contribute to decreased cytidine analog half-life and likely contributes to worse outcomes with decitabine therapy [9, 21]. However, in our study, this ratio did not differ significantly between responders and non-responders. It is conceivable that this discrepancy reflects differences in drug metabolism among diverse racial groups.

Unlike traditional cytotoxic therapies that induce rapid responses in MDS (mostly after one or two cycles) [22, 23], decitabine has a different pattern, in which a response is rare after one cycle but improves over time [4, 24]. Our results showed that patients with higher *hENT1* expression achieved CR with fewer cycles of therapy than those with lower *hENT1* expression. We speculate that higher *hENT1* levels can result in higher intracellular concentrations for one dose, a subsequently improved drug efficacy for each cycle, and therefore fewer cycles to reach the best response.

The results also showed that high expression of *hENT1* predicted a prolonged survival in higher-risk MDS patients receiving decitabine treatment. Multivariate regression analysis confirmed that the *hENT1* expression level was an independent prognostic indicator in higher risk MDS patients treated with decitabine, regardless of BM blast percentage, platelet count or hemoglobin level. The prolonged survival in patients with high *hENT1* expression may result from a better response compared with patients with low *hENT1*. Therefore, besides the prognosis predicting system such as IPSS/IPSS-Revision (IPSS-R) [25]/WHO prognostic scoring system (WPSS) [26], *hENT1* expression level could serve as a complementary prognosis predictor for higher-risk MDS patients treated with decitabine.

The in vitro study showed that knockdown of *hENT1* weakened the hypomethylation effect of decitabine treatment in an MDS cell line. Moreover, the biggest gap in the demethylation effect between the two groups was at a decitabine treatment concentration of 0.5 µmol/l, while the effect tended to close in the control group with increasing decitabine concentrations. Therefore, *hENT1* expression levels appear to affect the decitabine efficacy at low concentrations. Increasing decitabine to a final concentration of 5 µM resulted in no difference between the hENT1-knockdown group and controls. Therefore, for MDS patients with low *hENT1* expression, increasing the dosage of decitabine may be a good choice to achieve a clinical response. Drugs targeting to increase expression of *hENT1* may reduce the primary resistance to decitabine and increase the response rate. Notably, other genes involved in the transcriptional down-regulation of *hENT1* expression may also affect the response to decitabine. The *hENT1* promoter region contains consensus sequences for several transcription factors, including the C/EBP homologous protein (CHOP), a member of the CCAAT/enhancer binding protein family [27, 28]. The CHOP may transcriptional regulate the expression of *hENT1* and thus affect the response to decitabine, which will be evaluated in our later study.

Nevertheless, the *hENT1* expression level is not the only factor that can predict response to decitabine. The expression of multidrug resistant protein 1 (MRP1) [29] may result to resistance to decitabine in MDS patients, which may partly account for the no response of the drug. Other factors including cytogenetic changes (monosomy 7 or complex karyotypes) [4, 20], and mutations in genes such as *TET2* [30] and *DNMT3a* [31], have also been documented. In the future, analysis of *hENT1* expression combined with other factors can serve as an effective response prediction system for decitabine therapy in MDS, which will benefit more patients.

## Conclusions

In summary, high expression of *hENT1* predicts a good response to decitabine and prolonged survival in higher-risk MDS patients treated with decitabine. Knockdown of *hENT1* expression weakens the demethylation effect of decitabine. The *hENT1* expression level could serve as an effective response predictor in MDS patients treated with decitabine.

## Additional file

10.1186/s12967-016-0817-9 HENT1 expression level increased significantly in patients achieved mCR (a) or HI (b) when compared to that in NR patients, while expression levels of hENT2, DCK, and CDA was not significantly different between mCR (a) or HI (b) patients and NR.

## Abbreviations

CCR: cytogenetic complete response
CDA: cytidine deaminase
CMML: chronic myelomonocytic leukemia
CR: complete response
DCK: deoxycytidine kinase
FAB: French–American–British
hENT1: human equilibrative nucleoside transporter 1
IPSS: international Prognostic Scoring System
LINE1: long interspersed nucleotide elements 1
MDS: myelodysplastic syndrome
OR: overall response
Q-PCR: quantitative real-time PCR
RAEB-1: refractory anemia with excess blasts-1
RAEB-2: refractory anemia with excess blasts-2
RAEB-t: refractory anemia with excess of blasts in transformation
RAS: refractory anemia with ringed sideroblasts
RCMD: refractory cytopenia with multilineage dysplasia
WHO: World Health Organization
