# Supplementary material for: High expression of the human equilibrative nucleoside transporter 1 gene predicts a good response to decitabine in patients with myelodysplastic syndrome
Source: J Transl Med. 2016 Mar 5;14:66. doi: 10.1186/s12967-016-0817-9 (PMC4779250; doi:10.1186/s12967-016-0817-9)
Supplement: Supplementary file 1 — 10.1186/s12967-016-0817-9 HENT1 expression level increased significantly in patients achieved mCR (a) or HI (b) when compared to that in NR patients, while expression levels of hENT2, DCK, and CDA was not significantly different between mCR (a) or HI (b) patients and NR. [file 12967_2016_817_MOESM1_ESM.docx]

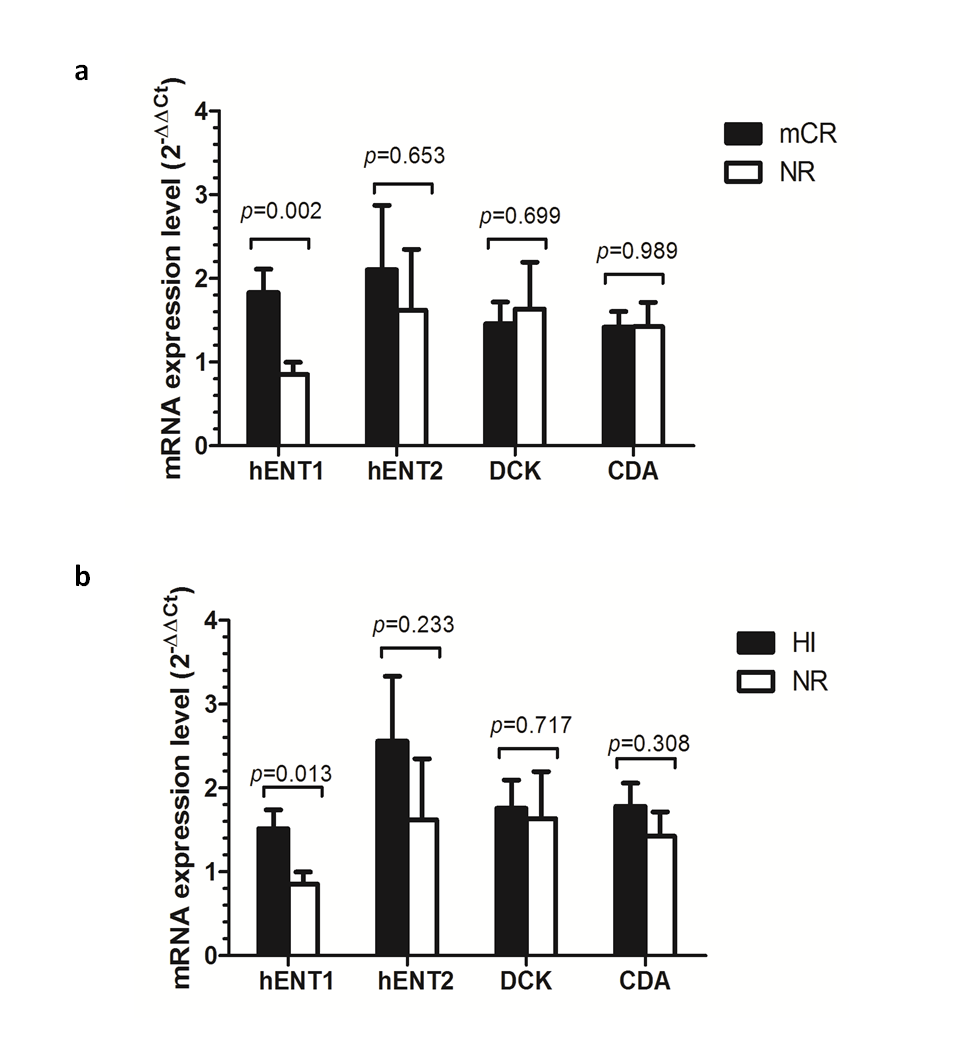


Supplementary 1 *HENT1* expression level increased significantly in patients achieved mCR (a) or HI (b) when compared to that in NR patients, while expression levels of *hENT2*, *DCK*, and *CDA* was not significantly different between mCR (a) or HI (b) patients and NR.
